# Comparison and prediction of pullout strength of conical and cylindrical pedicle screws within synthetic bone

**DOI:** 10.1186/1471-2474-10-44

**Published:** 2009-04-30

**Authors:** Wen-Chi Tsai, Po-Quang Chen, Tung-Wu Lu, Shing-Sheng Wu, Kao-Shang Shih, Shang-Chih Lin

**Affiliations:** 1 Institute of Biomedical Engineering, National Taiwan University, Taipei, Taiwan, ROC; 2 Department of Orthopedic Surgery, Shuang-Ho Hospital, Taipei Medical University, Tapei, Taiwan, ROC; 3 Department of Orthopedic Surgery, Ming-Sheng General Hospital, Tau-Yuang, Taiwan, ROC; 4 Division of Orthopedic Surgery, Department of Surgery, Far Eastern Memorial Hospital, Taipei, Taiwan, ROC; 5 Department of Mechanical Engineering, National Central University, Taoyuan, Taiwan, ROC; 6 Institute of Biomedical Engineering, National Central University, Taoyuan, Taiwan, ROC

## Abstract

**Background:**

This study was designed to derive the theoretical formulae to predict the pullout strength of pedicle screws with an inconstant outer and/or inner diameter distribution (conical screws). For the transpedicular fixation, one of the failure modes is the screw loosening from the vertebral bone. Hence, various kinds of pedicle screws have been evaluated to measure the pullout strength using synthetic and cadaveric bone as specimens. In the literature, the Chapman's formula has been widely proposed to predict the pullout strength of screws with constant outer and inner diameters (cylindrical screws).

**Methods:**

This study formulated the pullout strength of the conical and cylindrical screws as the functions of material, screw, and surgery factors. The predicted pullout strength of each screw was compared to the experimentally measured data. Synthetic bones were used to standardize the material properties of the specimen and provide observation of the loosening mechanism of the bone/screw construct.

**Results:**

The predicted data from the new formulae were better correlated with the mean pullout strength of both the cylindrical and conical screws within an average error of 5.0% and *R*^2 ^= 0.93. On the other hand, the average error and *R*^2 ^value of the literature formula were as high as -32.3% and -0.26, respectively.

**Conclusion:**

The pullout strength of the pedicle screws was the functions of bone strength, screw design, and pilot hole. The close correlation between the measured and predicted pullout strength validated the value of the new formulae, so as avoid repeating experimental tests.

## Background

Transpedicular screw fixation has been extensively used for the treatment of instability due to degenerative disorders, trauma, tumor metastasis, and deformity correction. It provides immediate stability, enhances bony fusion, corrects deformity, and preserves the anatomic profile. However, breakage and the loosening of pedicle screws from the vertebral bone are two main clinical concerns [1].

In the literature, synthetic bones were selected for the measurement of the screw resistance to loosening [2-16]. Chapman *et al*. [4] used an analytical formula to predict the pullout strength of cancellous and/or cortical screws inserted into the synthetic bone. The Chapman's formula was confirmed by some reports to be valuable because of the close correlation between the predicted and the measured pullout strength [3,4,7,11,17]. However, pedicle screws of the spine usually have different profiles. The Chapman's formula was derived for the cancellous/cortical screw, and the thread design was different from the pedicle screw with conically distributed inner and/or outer diameters. Furthermore, the effects of the bone removal by pre-drilling a pilot hole and subsequently squeezing the bone chip into the thread surroundings were not considered in the Chapman's formula.

The purposes of the current study were threefold. Firstly, the pullout strength of six cylindrical and conical pedicle screws within the synthetic bone were measured and compared. Then, the measured pullout strength data were tested to prove the accuracy of the Chapman's formula in predicting the pullout strength of various pedicle screws. Finally, special emphasis was put on deriving a new formula that takes into consideration the effects of the pilot hole and the squeezed bone chip at the thread surroundings.

## Methods

**Figure 1:**
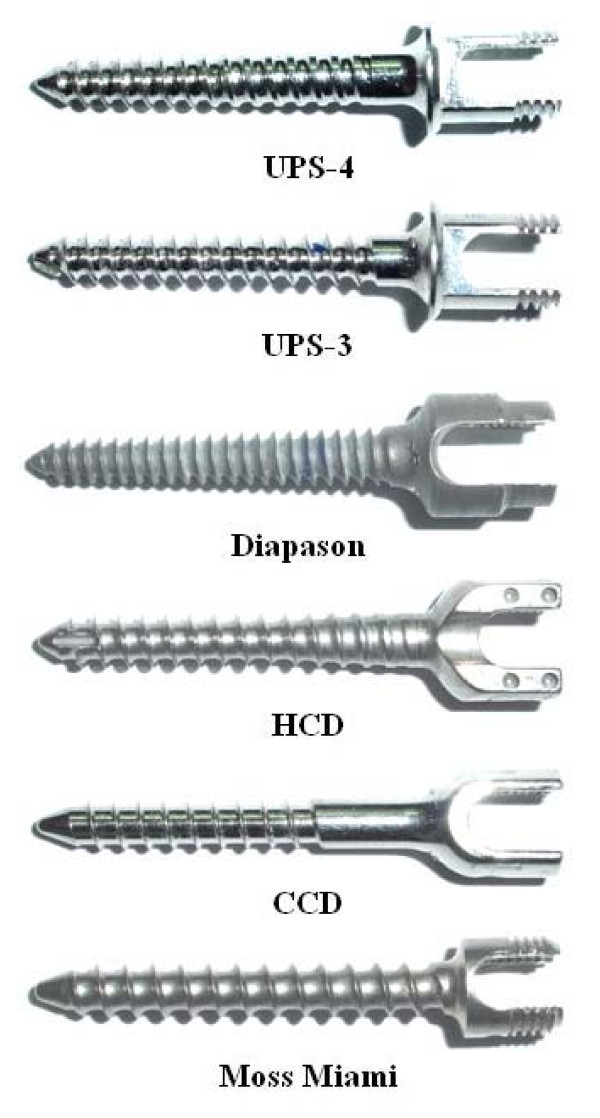
**The six Pedicle Screws used in this study**. Three pedicle screws (UPS-4, Diapason, and HCD) have a conical inner core, while the other three (UPS-3, CCD and Moss Miami) have a cylindrical core.

In this study, six pedicle screws with distinctly different thread designs were used to evaluate their pullout strength within synthetic bone. Accordingly, the measured pullout data were compared with the predicted values from both the Chapman's and the new formulae. Figure 1 shows the appearance of those six pedicle screws: UPS-3, UPS-4 (both by Aaxter Co, Taipei, Taiwan), Diapason (Dimson/Stryker, Bordeaux, France), Horizon CD (abr. HCD, Sofamor/Danek, Memphis, USA, Compact CD (abr. CCD, Sofamor, France), and Moss-Miami (Depuy/Johnson Johnson, New Jersey USA). The inner core profiles of the UPS-4, Diapason, and HCD are conical in shape, while those of the UPS-3, CCD, and Moss-Miami are cylindrical in shape (Figure 1). The outer thread profile of the Diapason screw is conical, while that of the others is cylindrical. The Moss-Miami has the widest, and the Diapason has the smallest pitch. The details of the geometrical parameters of these six pedicle screws are listed in Table 1.

**Table 1:** The design parameters and the equivalent diameter functions of the six pedicle screws.

**Pedicle Screw**	**Threaded Length**	**Outer Diameter**	**Inner Diameter**	**Screw Pitch**	**Equivalent Diameter Function** (0≤*x*≤*30*)
**UPS-4**	37	6.0 *(0≤ x≤ 37)*	3.8 (*x *= 0) 5.4 (*x *= 30)	2.4	*D*_*o*_*(x) = 6.0*

**UPS-3**	37	6.0 *(0≤ x≤ 37)*	4.0 *(0≤ x≤ 37)*	2.5	*D*_*o*_*(x) = 6.0* *D*_*i*_*(x) = 4.0*

**Diapason**	40	4.6 (*x *= 0) 6.0 (*x *= 30)	2.6 (*x *= 0) 5.0 (*x *= 30)	1.75	

**HCD**	40	6.0 *(0≤ x≤ 40)*	4.2 *(0≤ x≤ 30)* 5.6 (*x *= 40)	2.7	*D*_*o*_*(x) = 6.0* *D*_*i*_*(x) = 4.2*

**CCD**	30	6.0 *(0≤ x≤ 30)*	4.5 *(0≤ x≤ 30)*	2.8	*D*_*o*_*(x) = 6.0* *D*_*i*_*(x) = 2.8*

**Moss-Miami**	35.5	6.0 *(0≤ x≤ 35.5)*	4.0 *(0≤ x≤ 35.5)*	2.9	*D*_*o*_*(x) = 6.0* *D*_*i*_*(x) = 2.9*

Unit: mm.

Synthetic bone made from polyurethane foam was used as the testing specimen for its consistent and homogeneous structural properties (Wuzhou Co, Taipei, Taiwan). The screw was inserted perpendicularly into the testing block following pre-drill pilot holes preparation. Pilot holes were prepared with a 3.2-mm drill bit, which was smaller than the inner diameter of the screw at the screw tip, except for the Diapason. All screws were engaged into the synthetic bones with a consistent 30-mm thread length. Tapping was not used during screw insertion.

**Figure 2:**
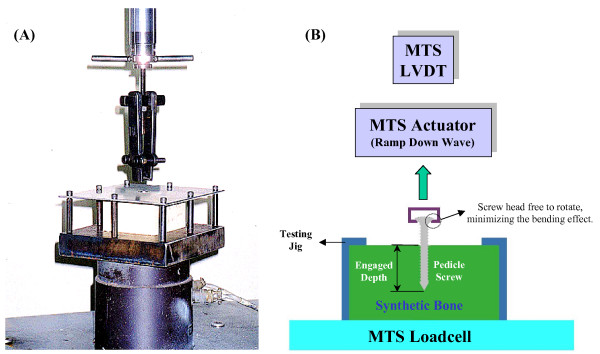
**The setup of testing in the MTS machine, jig assembly, and synthetic bone**. The synthetic bone (white color) was encased in the jig, and the screw head was firmly attached to the grip on the top.

An axial pullout test was performed using a servohydraulic-testing machine (MTS 858, Minneapolis, MN, USA). The pedicle screws were inserted perpendicular to the surface at different sites of the synthetic bones. Each screw underwent six trials. The synthetic bone was mounted on the testing jig, and then a monotonic tensile load with an actuator speed of 5 mm/min was applied along the axis of the screw [18]. The jig assembly consisted of a simple fixture attached to the platform of the testing machine and the screw-to-crosshead coupling fixture. The screw-to-crosshead coupling fixture permitted application of a pure tensile force without inducing a bending moment. The measured maximum load prior to free displacement of the screw from the synthetic bone was defined as the pullout strength of the screw. The setup of the testing machine, jig assembly, and synthetic bone was schematically shown in Figure 2.

In the literature, Chapman *et al*. [4] propose an analytical formula to predict the pullout strength of a cancellous screw within synthetic bone. They assumed the tearing failure of a screw/bone construct along an ideally cylindrical surface with an equivalent cross-sectional area by introducing a thread shape factor (TSF). They did not consider that the conical thread pattern, pilot hole, and amount of squeezed bone may modify the ultimate shear strength of the synthetic bone. The Chapman's formula has the following form:



The symbols in the formula were respectively denoted as: *F*_*pullout *_= predicted pullout strength (N), *S*_*shear *_= ultimate shear strength of synthetic bone (MPa), *L *= length of thread engagement in synthetic bone (mm), *D*_*o *_= thread outer diameter (mm), *d *= thread depth (mm), and *p *= thread pitch (mm). The equivalent cross-sectional area of the Chapman's formula equals  and, *that is*, the thread shape factor is .

**Figure 3:**
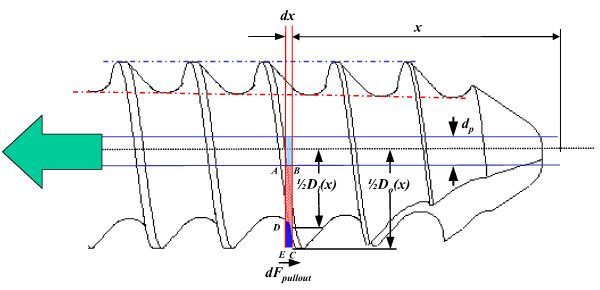
**The mechanism of the Integral formula to predict the pullout strength of the pedicle screw with variable distributions of outer and inner diameters (*i.e*., conical or cylindrical screw)**. The diagram shows the insertion of a conical shaped screw into the synthetic bone. The bone chips within the region *ABCD *was assumed to be squeezed into the region *CDE*. See the text for the detailed meaning of the symbols.



In the literature, Asnis *et al*. [3] expressed the ultimate shear strength, *S*_*shear*_, of synthetic bone as a function of its density, *ρ*, as follows:



The symbols, *a *and *b*, are the material constants of the synthetic bone. From the surgical and experimental viewpoints, it is necessary to drill a pilot hole as a guide for easy screw insertion. Consequently, during screw insertion into the pilot hole, the screw squeezes the synthetic bone away, and the shear strength of the surrounding synthetic bone must be modified and calculated. In the real situation, the cutting flutes at screw tip and the interfacial friction between bone and thread squeeze the bone chips towards to the uncertain regions, depends on the screw design, bone property, and insertion process. For simplification, the synthetic bone mass within the region *ABCD *was assumed to be uniformly squeezed into the region *CDE *(Figure 3). Then the modified ultimate shear strength, , of the squeezed synthetic bone can be defined as below:

The definition of the symbol is respectively as: *D*_*o*_*(x) *= outer-diameter function along the screw shaft (mm), *D*_*i*_*(x) *= inner-diameter function along the screw shaft (mm), and *d*_*p *_= diameter of the pilot hole (mm). For the six screws used in this study, the functions of *D*_*o*_*(x) *and *D*_*i*_*(x) *within *0 ≤ x ≤ 30 *mm are given in Table 1, and schematically shown in Figure 3.

In Figure 3, the differential pullout strength, *dF*_*pullout*_, was calculated in a manner similar to the Chapman's assumption. The thread shape factor was also modified to account for variable thread distributions.



The total pullout strength *F*_*pullout *_can integrate the differential pullout strength along length of the thread engaged in the synthetic bone.



For the practical purpose, the integral formula can be further simplified by reducing the originally conical screw to an equivalently cylindrical screw. Consequently, by introducing two parameters, *m *and *n*, the integral formula has the following form:





The terms *D*_*o*-*equ *_and *D*_*i*-*equ *_were respectively the equivalent outer and inner diameters. The symbol *d*_*equ *_was the equivalent thread depth (mm). The term  of the modified Chapman's formula was the factor that incorporated the effects of the ratio of the inner and outer diameters, squeezed bone mass, and pilot hole into calculating pullout strength. In this study, within the 30-mm engaged depth, the inner core and outer thread peaks of UPS-3, CCD, HCD, and Moss-Miami have nearly constant diameters (Figure 1 and Table 1). Hence, the equivalent outer and inner diameters are constant along the screw shaft. However, the equivalent outer and inner diameters of the conical screws, UPS-4 and Diapason, are calculated as: *D*_*o-equ *_= *D*_*o *_(*x*) and *D*_*i-equ *_= *D*_*i *_(*x*) for three cases: *x *= 7.5 mm, 15.0 mm, and 22.5 mm. According to the ASTM testing standard [19], the shear strength and the material constant of the polyurethane foam used in this study were experimentally evaluated and have the value of 290 MPa and 0.84, respectively. All the predicted pullout strengths using the Chapman's, the Integral, and the modified Chapman's formulae were calculated by the software Mathematica, Ed. 5 (Wolfram Research, Champaign, IL).

For pullout tests, each pedicle screw was tested six times, and the mean and one standard deviation were calculated. The statistical analyses were performed using analysis of variance with multiple comparisons between groups (Student-Newman-Keuls test) to test the significance of the variation between six pedicle screws. A *p *value less than 0.05 was considered to be statistically significant. For the analytical formulae, the coefficient of determination, *R*^2^, between the measured and predicted pullout strengths was calculated to check their correlation.

## Results

### Measured Pullout Strength

**Figure 4:**
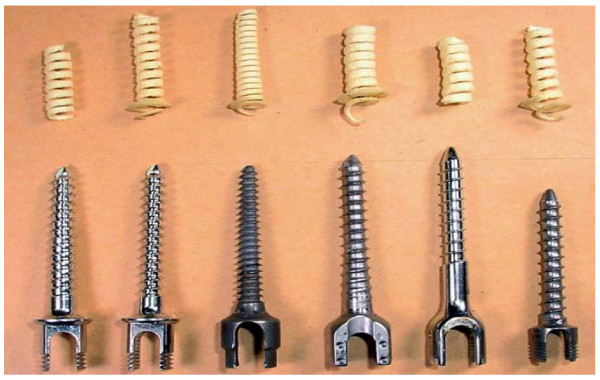
**The spiral of bone chips**. The spirals of bone chips were peeled from the surface of the screw threads.

Figure 4 shows the withdrawn bone chips surrounding the thread surface. Special emphasis was put on the observation that the outer surface of the bone chip peeled off the valleys of the screw thread forming a cylindrical (UPS-4, UPS-3, HCD, CCD, and Moss-Miami) or conical (Diapason) spiral. This finding does not support the prerequisite of the Chapman's formula that the tearing failure of the screw/bone construct is along an ideally cylindrical surface.

The pullout strength of each pedicle screw (mean value ± one standard deviation) was 1904 ± 72 N for UPS-4, 1600 ± 53 N for UPS-3, 1568 ± 46 N for Diapason, 1583 ± 59 N for HCD, 1610 ± 33 N for CCD, and 1570 ± 100 N for Moss-Miami (Table 2). The highest pullout strength was the UPS-4 screw, and there was no significant difference in pullout strength among the other screw designs.

**Table 2:** The measured and predicted pullout strengths of the six pedicle screws.

**Group**		**Pullout Strength** (mean value ± one standard deviation)						***R*^2 ^Value**
			
		**UPS-4**	**UPS-3**	**Diapason**	**HCD**	**CCD**	**Moss-Miami**	
**Experiment**		1904 ± 72	1600 ± 53	1568 ± 46	1583 ± 59	1610 ± 33	1570 ± 100	---

**Chapman's Formula**	1/4*L*	1175	1198	1067	1035	1073	1146	0.40
	1/2*L*	1096	1198	1082	1035	1073	1146	-0.26
	3/4*L*	1017	1198	1090	1035	1073	1146	-0.71

**Integral Formula**		1861	1483	1411	1509	1636	1481	0.93

**Modified Chapman's Formula**	1/4*L*	1561	1482	1067	1509	1622	1418	0.38
	1/2*L*	1742	1482	1356	1509	1622	1418	0.83
	3/4*L*	2079	1482	1725	1509	1622	1418	0.88

There are three types of defining the equivalent outer and inner diameter of the UPS-4 and Diapason screws in the Chapman's and modified Chapman's formulae. Unit: N.

Table 2 lists the measured and predicted pullout strengths of six pedicle screws using three formulae in three equivalent diameter cases. The prediction error for each screw between the experimental and the Chapman's data was -38.3% (UPS-4), -25.1% (UPS-3), -40.0% (Diapason), -34.6% (HCD), -33.4% (CCD), -27.0% (Moss Miami) for the *x *= 7.5 mm case, -42.4% (UPS-4), -25.1% (UPS-3), -31.0% (Diapason), -34.6% (HCD), -33.4% (CCD), -27.0% (Moss Miami) for the *x *= 15.0 mm case, and -46.6% (UPS-4), -25.1% (UPS-3), -30.5% (Diapason), -34.6% (HCD), -33.4% (CCD), -27.0% (Moss Miami) for the *x *= 22.5 mm case. The minus values of the prediction error revealed that the Chapman's formula underestimates the pullout strength of even the cylindrical pedicle screw.

### Predicted Pullout Strength

**Figure 5:**
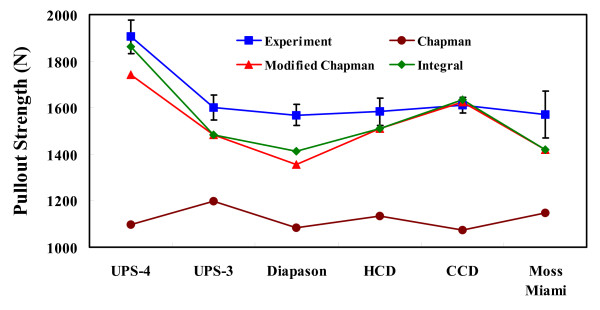
**The measured and predicted pullout strength of six pedicle screws**. One standard deviation is used as the error bar of the experimental data. The 0.5 value of the equivalent diameter ratio is used in calculating the Chapman's and Lin's formulae. It is obvious that the predicted pullout strength of each screw using the new formula was closer to those under testing, while the calculated data from the Chapman's formula were very variable and could not match the experimental data.

The predicted pullout strengths using the Chapman's, Integral, and modified Chapman's formulae for each screw are listed in Table 2 and Figure 5. For the Chapman's formula, the *R*^2 ^value between the predicted and measured pullout strength for three cases (*x *= 7.5 mm, 15.0 mm, and 22.5 mm) was 0.40, -0.26, and -0.71, respectively. From Table 2 and Figure 5, the predicted pullout strength using the modified Chapman's formula was more correlated with the experimental data (*R*^2 ^values: 0.38, 0.83, and 0.88). By contrast, the predicted result using the Integral formula showed the largest *R*^2 ^value (= 0.93).

For the screws with constant inner and outer diameters within the engaged length, such as the UPS-3, HCD, CCD, and Moss-Miami, the predicted pullout strengths using the modified Chapman's and Integral formulae was the same as that shown in Table 2 and Figure 5. For the conical screw, such as the UPS-4 and Diapason, the Integral formula prediction had a more accurate result than that of the modified Chapman's formula.

## Discussion

In a report by Esses *et al*. [20], of the 617 cases using various pedicle screws, the rate of screw breakage was 2.9%, and that of screw loosening was 0.8%. Yuan *et al*. [21] reported that screw breakage was observed in 2.6% and screw loosening in 2.8% of the 2153 patients treated for degenerative spondylolisthesis. The rate of screw breakage ranged from 6% to 21%, while the loosening rate ranged from 18% to 27% [1,21-23]. It is mandatory that the implants are rigid enough for fixation of the spinal segments. However, if screw loosening or breakage occurs before rigid fusion is achieved; it will increase the likelihood of non-union with subsequent morbidity. Therefore, some kinds of predictive guidelines to assure the pullout strength of pedicle screws are necessary before fabrication and clinical usage.

Synthetic bone has been extensively used in the biomechanical evaluation of bone screws [2-16]. In this study, the advantageously consistent property of the solid polyurethane foam was reflected in the relatively small standard deviations in pullout strength, which were less than 7% of mean values (Table 2). The continuous bone chips stripped off the screw thread provide clear observation and insight into the loosening mechanism of the bone/screw construct (Figure 4). In this study, the length of the threaded portion was different for six pedicle screws (Figure 1). During testing, the engaged length of all screws within the synthetic bone was consistently 30 mm. The measured pullout strength in this study cannot be directly used as a basis for comparison of the resistance to loosening of the six pedicle screws fully immersed in the vertebral bone. The experimental comparison in pullout strength of the six pedicle screws was only to validate the reported mathematical formula and provided a basis for deriving a new formula with more accurate prediction in the early stage of screw design.

The prerequisite of the Chapman's formula is that the screw is pulled out from the synthetic bone along an ideally cylindrical surface. This assumption is straightforward, as shown in Figure 4. The outer surface of the bone chip peeled off the UPS-4, UPS-3, HCD, CCD, and Moss-Miami screws formed a spiral with a cylindrical outer diameter. For the Diapason screw, the spiral of bone chip has the appearance of linearly variable outer and inner diameters. However, the pullout strength in the Chapman prediction was proportional to the cross-sectional area of the assumed shearing surface. Hence, the discrepancy between the experimental observation and the Chapman's assumption about the tearing surface of the bone/screw construct may induce a prediction error for the pullout strength of conical screws, such as the Diapason.

Table 2 lists the measured and predicted pullout strengths of six pedicle screws using three formulae in three equivalent diameter cases. Even for the UPS-3, HCD, CCD, and Moss-Miami screws with a cylindrical chip spiral, the average prediction error of the three cases (*x *= 7.5 mm, 15.0 mm, and 22.5 mm) was still around -30%. The average prediction error of the UPS-4 screw with cylindrical chip spiral is even up to -42.4%. These findings regarding cylindrical pedicle screws differed from those of previous reports, which claimed that a strong correlation did exist between the Chapman prediction and experimental data [3,4,7,11,17]. Hence, the factor of assuming the tearing surface between the bone and screw, alone, cannot account for the quite large inaccuracy of the Chapman prediction. Consequently, the ratio of inner to outer diameter and the effect of the squeezed bone chip and pilot hole were taken into consideration to improve the prediction accuracy of the analytical formula.

Figure 3 shows that the Integral formula assumes the tearing surface of the bone/screw construct occurring at the surface formed by the thread peaks along the screw shaft. This is consistent with the bone chip spirals as shown in Figure 4. As aforementioned, the modifications in the geometry of the tearing bone chip alone may not accurately predict the pullout strength of various bone screws. Hence, the term  in the Integral formula was added to incorporate the effects of the pilot hole, squeezed bone chip, and ratio of inner to outer diameter into the prediction of pullout strength. The exponent *b *in the Integral formula was denoted as a material factor in response to the screw insertion-induced change in shear strength of the synthetic bone. The pilot-hole factor, *d*_*p*_, was used to consider the removal of the synthetic bone during pre-drilling of the pilot hole (Figure 3). The simplification of the base expression, , resulted in another thread shape factor , which is the ratio of the thread inner to outer diameter. In the modified Chapman's formula, the term  was further simplified to be , which can be interpreted as a thread ratio-, pilot hole-, and material-induced factor of the Chapman's formula. For the cylindrical screw, the Integral and modified Chapman's formulae were theoretically identical to each other.

In the literature, the pullout strength of the bone screw has been biomechanically proven to be a function of the screw design (outer/inner diameter, thread pitch, flank angle, threaded length, cutting flute, and cannulated/noncannulated), screw orientation, screw-insertion depth, bone-mineral density, pilot hole, bone morphology, surface coating, and loading type [1-7,13,15,16,23-25]. In general, those studies revealed that 1) outer diameter is an important determinant of pullout strength in a roughly linear manner, 2) pitch is important with a finer thread giving greater purchase, 3) flank angles significantly affect the holding power of the inserted screw, 4) inner diameter and the ratio of inner to outer diameter has a small but significant effect on the pullout strength, and 5) the pilot hole has a significant influence on the pullout strength with non-pilot hole groups, resulting in higher holding power. However, the Chapman's formula was derived for the cylindrically cancellous/cortical screw with the triangular thread shape. This research work was focused on the modification of the Chapman's formula, and, only the effects of tapering profile, pre-drilling hole, and squeezing chip were formulated to predict the pullout strengths of the six varities of pedicle screws.

**Figure 6:**
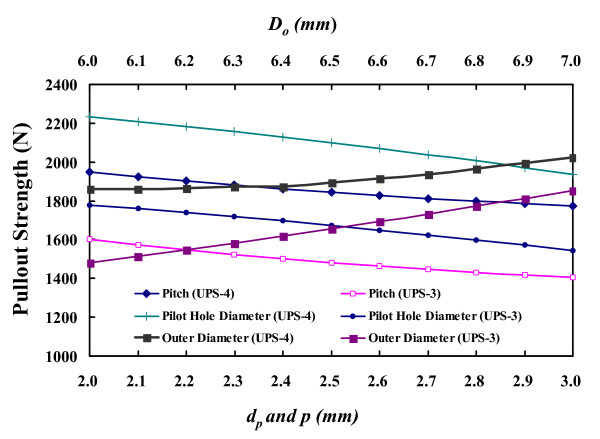
**The parametric analyses of the parameters of screw shape and pilot hole**. The parametric analyses of the parameters of screw shape (*D*_*o *_and *p*) and pilot hole (*d*_*o*_) in the new formulae.

The influence of the screw profile and pilot hole on the predicted pullout strength can be estimated from the Integral and modified Chapman's formulae and shown in Figure 6. For the cylindrical (UPS-3) and conical (UPS-4) screws, the increase in *D*_*o *_stiffens the screw resistance to axial loosening from the synthetic bone (Figure 6). The change in outer diameter from 6.0 to 7.0 mm increases the pullout strength by 8.8% and 25.0% for the UPS-4 and UPS-3 screws, respectively. The tendency of the cylindrical screw toward an increase in pullout strength is more significant than that of the conical one. Figure 6 also shows a similar result with the aforementioned finding that the finer thread pitch results in greater pullout strength. The change in *p *from 2.0 to 3.0 mm decreased 9.0% and 12.2% of pullout strength for the UPS-4 and UPS-3 screw, respectively. Both new formulae predicted the increase in *d*_*p *_would decrease the amount of the bone chip squeezed into the thread surroundings (region *CDE*), thus decreasing the pullout strength. The decrease in pullout strength of both the UPS-4 and UPS-3 screws was the same (13.3%). However, if a great amount of bone chip was squeezed into the very narrow region *CDE*, and thus induced bony micro-fracture, the applicability of the new formulae may not be suitable. In this condition, finite element analysis may be an alternative to predict the pullout strength of the screws [10,20,25,26].

Cutting flute at the screw tip facilitates insertion, but this region of the screw has less pullout strength than the fully-threaded region. Theoretically, the shear strength, , of the squeezed bone chip at the thread surroundings is closely related to the tapping function of cutting flute. Yerby *et al*. [27] have also biomechanically shown that cutting a flute design significantly influences the mean insertion torque and pullout strength. However, the effect of this cutting-flute factor on the shear strength of synthetic bone in the region *CDE *was complicated and not considered in the new formulae (Figure 3).

In the clinical situation, the inserted regions of the pedicle screw include the vertebral body and the posterior element (pedicle). The vertebral body consists of the cancellous bone with the porous structure in nature. The structure of the posterior element is denser and stiffer than that of the vertebral body. Consequently, the polyurethane foam used in this study was to simulate the hybrid of the vertebral bone and posterior element. In addition, the Asnis's formula cited in this study is more suitable for the polyurethane foam with the lower porosity than the cancellous bone. The applicability of the new formula should be further investigated for predicting the pullout strength of the inserted screw within the cancellous bone. For example, the ultimate shear strength of the squeezing effect bone chips with the higher porosity might be reformulated.

In the literature, a great number of studies have attempted to show experimentally that the conical design of the screw profile increases the pullout strength, and the increasing degree depends on the test medium and design [23,26-28]. The current testing study also demonstrated that the conical-shaped UPS-4 screw had higher pullout strength than its cylindrical-shaped counterpart, UPS-3, (Table 2 and Figure 3). However, except for the UPS-4 screw, there was no significant difference in pullout strength between the Diapason and the other cylindrical-shaped screws. The Integral and modified Chapman's formulae also predicted similar results (Table 2 and Figure 5). This meant that the pullout strength of the pedicle screw was the result of a number of varying parameters, not only the conical- and cylindrical-shaped profile. The isolation of the related parameters was necessary to study the influence of one particular parameter on the pullout strength of the commercially available screws.

The coefficient of determination between the measured and predicted pullout strength has been used as an indicator to confirm the accuracy of the Chapman's formula [3,6,7,11]. However, as shown in Table 2, the *R*^2 ^values between the predicted and measured pullout for three cases (*x *= 7.5 mm, 15.0 mm, and 22.5 mm) were 0.40, -0.26, and -0.71, respectively. By contrast, the 0.83 and 0.88 of the *R*^2 ^value in the *x *= 15.0 mm and 22.5 mm cases proved that the modified Chapman's formula was quite well correlated with the experimental data. In particular, the predicted result using the Integral formula had the highest *R*^2 ^value (= 0.93). For the cylindrical screws, such as the UPS-3, HCD, CCD, and Moss-Miami, the predicted pullout strengths by the modified Chapman's and Integral formulae were the same as shown in Table 2 and Figure 5. For the conical screws, such as the UPS-4 and Diapason, the Integral formula had the best predicted value.

## Conclusion

This study was designed to derive the analytical formula for predicting the pullout strength of both conical and cylindrical pedicle screws. The new formula is a function of material (shear strength of synthetic bone), screw (diameter and pitch), and surgery (pilot hole) factors. The strong correlation between the measured and the predicted pullout strength validated the value of the new formula. The usage of the new formulae can eliminate the need for costly and time-consuming repeated mechanical testing. However, the newly derived formulae were only validated by the synthetic bones. In the future, the detailed investigation and validation about the screw-bone interfaces should be studied by the finite-element method and biomechanical evaluation using cadaver specimens.

## Competing interests

The authors declare that they have no competing interests.

## Authors' contributions

WCT, PQC, and KSS conceived of the study, participated in the design of the study and performed the data analyses. SCL formulated the model and drafted the manuscript with the help of TWL and SSW. All authors carried out the experiments, read, and approved the final manuscript.

## Pre-publication history

The pre-publication history for this paper can be accessed here:


